# The Activity of the Durum Wheat (*Triticum durum* L.) Catalase 1 (TdCAT1) Is Modulated by Calmodulin

**DOI:** 10.3390/antiox11081483

**Published:** 2022-07-29

**Authors:** Mouna Ghorbel, Kaouthar Feki, Sana Tounsi, Najla Haddaji, Moez Hanin, Faiçal Brini

**Affiliations:** 1 Biology Department, Faculty of Science, University of Hail, Hai’l P.O. Box 2440, Saudi Arabia; m.ghorbel@uoh.edu.sa (M.G.); n.haddaji@uoh.edu.sa (N.H.); 2 Laboratory of Biotechnology and Plant Improvement, Center of Biotechnology of Sfax, P.O. Box 1177, Sfax 3018, Tunisia; sana.tounsi@cbs.rnrt.tn; 3 Laboratory of Legumes and Sustainable Agrostems (L2AD), Center of Biotechnology of Bordj Cedria, P.O. Box 901, Hammam Lif 2050, Tunisia; kaouthar.feki@cbbc.rnrt.tn; 4 Unité de Génomique Fonctionnelle et Physiologie des Plantes, Département de Biologie, Institut Supérieur de Biotechnologie, Université de Sfax, BP “1175”, Sfax 3038, Tunisia; moez.hanin@isbs.rnu.tn

**Keywords:** Ca^2+^, calmodulin, calmodulin binding domain, catalase, durum wheat, ROS

## Abstract

Plant catalases (CAT) are involved in the cellular scavenging of the reactive oxygen species during developmental processes and in response to abiotic and biotic stresses. However, little is known about the regulation of the CAT activity to ensure efficient antioxidant function. Using bioinformatic analyses, we showed that durum wheat catalase 1 (TdCAT1) harbors highly conserved cation-binding and calmodulin binding (CaMBD) domains which are localized at different positions of the protein. As a result, the catalytic activity of TdCAT1 is enhanced in vitro by the divalent cations Mn^2+^ and Fe^2+^ and to a lesser extent by Cu^2+^, Zn^2+^, and Mg^2+^. Moreover, the GST-pull down assays performed here revealed that TdCAT1 bind to the wheat CaM (TdCaM1.3) in a Ca^2+^-independent manner. Furthermore, the TdCaM1.3/Ca^2+^ complex is stimulated in a CaM-dose-dependent manner by the catalytic activity of TdCAT1, which is further increased in the presence of Mn^2+^ cations. The catalase activity of TdCAT1 is enhanced by various divalent cations and TdCaM1.3 in a Ca-dependent manner. Such effects are not reported so far and raise a possible role of CaM and cations in the function of CATs during cellular response to oxidative stress.

## 1. Introduction

Reactive oxygen species (ROS), such as singlet oxygen (^1^O_2_), hydrogen peroxide (H_2_O_2_), superoxide anion (O_2_^●−^), and hydroxyl radical (^−^OH), are toxic byproducts of the normal oxygen (O_2_) metabolism. In fact, they cause serious damage to essential macromolecules (proteins, lipids, and nucleic acid) by inducing oxidative stress [1]. On the other hand, ROS produced at low concentrations acts as a secondary messenger in plant cell response to different stresses such as water deficit, salinity, and extreme temperatures [1]. Previous studies revealed that those signaling molecules are crucial for maintaining normal cellular functions, including cell proliferation and differentiation, as well as stem cell maintenance [2]. They also trigger cell death as a necessary process for plant resistance to biotic and abiotic stresses [3,4]. Thus, cells must maintain a constant basal level of ROS to ensure ROS signaling by controlling the balance between the production and removal of ROS [5]. ROS scavenging pathway in plants is ensured by non-enzymatic and enzymatic antioxidants systems. Superoxide dismutases, peroxidases, and catalases are among the most important enzymatic antioxidants [6,7].

Catalase (CAT) is a tetrameric heme-containing enzyme that acts to remove the excessive H_2_O_2_ generated during developmental processes or by environmental stimuli into water and oxygen in all aerobic organisms [8]. Through its action, CAT plays a crucial role in plant growth, maturation, fruit ripening, postharvest events, and stress responses [9]. In higher plants, most catalases are reported to be localized in peroxisomes, glyoxysomes, and unspecialized peroxisomes of leaves, roots, and cotyledons [7,9,10].

Unlike animals, which harbor only one CAT encoding gene, plant genomes encode for multiple isozymes, and their numbers vary depending on the species [9]. For example, tobacco (*Nicotiana plumbaginifolia Viviani*), maize (*Zea mays*), *Arabidopsis thaliana*, and rice (*Oryza sativa*) genomes harbor three different genes, each encoding for a catalase isozyme [11]. However, there are two catalase genes in barley (*Hordeum vulgare*) and peach (*Prunus persica*) [12].

Several proteins are reported to interact with catalases, such as salt overly sensitive protein (SOS_2_) [13], nucleoside diphosphate kinase 1 (NDK1) [14] triple gene block protein 1 (TGBp1) [15], and LESION SIMULATING DISEASE1 (LSD1) [16], suggesting an interconnection between ROS status and various biotic and abiotic stress responses. In *Arabidopsis*, it has been demonstrated that the small heat shock protein Hsp14.6CII interacts with catalase AtCAT2 in the cytosol [17], as well as in the peroxisomes, and this interaction increases the catalytic activity of AtCAT2 in a NAC1 (a chaperone of catalase) dependent manner [18].

Calmodulins (CaMs) are small acidic proteins (148 aa) highly conserved that are in eukaryotic cells [19]. They act as the most relevant calcium sensors [19] that perceive transient changes in cytosolic Ca^2+^ levels [20] and participates in different cellular processes such as plant growth and responses to biotic and abiotic stresses [20,21]. CaM binds four Ca^2+^ ions with high affinity for calcium binding domains called EF-hand motifs [22,23] arranged in N- and C-terminal globular domains [24]. Upon binding to Ca^2+^, CaM changes conformation from a closed, Ca^2+^-free-state (apoCaM) to an extended Ca^2+^/CaM conformation. This conformational change allows the hydrophobic surfaces surrounded by negative charges to be exposed to target proteins with high affinity [19,24]. This structural flexibility allows CaM to regulate numerous protein targets implicated in a huge range of cellular responses to various signals such as cold, wind, wounding, pathogenic attacks, and also in gene regulation [25]. It is estimated that around 300 proteins can bind to CaMs in plants [25], such as the durum wheat pathogen-related protein (PR-1) [26]; MAP Kinase Phosphatase [27], transcription actors [28], and CATs as revealed in potato [29], *Arabidopsis* [30], and sweet potato [31].

In previous work, the durum wheat catalase TdCAT1 was shown to be involved in the tolerance to several abiotic stresses in yeast and *Arabidopsis* [32]. Sequence analysis revealed that TdCAT1 harbors a putative calmodulin-binding domain (CaMBD) that is localized at its C-terminal part (413–453 aa) [32]. This domain was reported to be essential for calmodulin binding and activation of some plant CATs in a calcium-dependent manner [30]. In this study, we provided in vitro evidence for the stimulation of TdCAT1 activity by divalent cations, especially Mn^2+^ and Fe^2+^. In addition, TdCAT1 binds via its CaMBD to TdCaM1.3 in a Ca^2+^-independent manner. Moreover, the TdCaM1.3/Ca^2+^ complex stimulates the catalase activity of TdCAT1 either alone or in the presence of Mn^2+^ and Fe^2+^. These data suggest the contribution of CaM and Mn^2+^ in the activation of TdCAT1, which may be valuable in enhancing plant stress tolerance [29,30].

## 2. Materials and Methods

### 2.1. Bioinformatic Analyses

Calmodulin binding domains were revealed by the calmodulin target database (http://calcium.uhnres.utoronto.ca/ctdb/ctdb/home.html (accessed in 12 December 2021)) [33]. Cation binding domains were investigated using uniprot database [34], Supfam databases (http://supfam.org/SUPERFAMILY/cgi–bin/align.cgi (accessed on 14 December 2021)) [35], and swiss model database, (https://swissmodel.expasy.org/interactive/T5XR77/models/ (accessed on 14 December 2021)) [36] for the identification of Mn^2+^/Mg^2+^, Ca^2+^, Zn^2+^/Cu^2+^ and Fe^2+^ binding domains respectively. HMMER database [37] was used for analyzing the functional domains present in catalase sequence. The catalytic parameters were calculated using the Michaelis–Menten Equation Calculator (https://www.mdapp.co/michaelis-menten-equation-calculator-431/ (accessed on 14 December 2021)).

### 2.2. Production and Purification of Recombinant TdCAT1 Proteins and Their Truncated Forms

In order to produce the recombinant proteins His_TdCAT1 and the different truncated forms [His_TdCAT_200_ (1–200 aa); His_TdCAT_295_ (1–295 aa); and His_TdCAT_460_ (1–460 aa)], each product was amplified by PCR with the Pfu Taq DNA polymerase and using the appropriate primers (Supplementary Table S1), digested by the appropriate restriction enzymes, *EcoR*I and *Xho*I, and cloned in-frame with a Histidine- tag into the pET28a expression vector (Novagen, Madison, WI, USA) into appropriate restriction sites. The same procedure was also conducted to produce the recombinant His_TdCaM1.3 (Accession N° MW057248). The product was amplified by PCR with the Pfu Taq DNA polymerase in the presence of the appropriate primers (Supplementary Table S1), containing *EcoR*I restriction sites, then digested and cloned in-frame with a Histidine-tag into the pET28a expression vectors. The resulting constructs (pHis_TdCAT1, pHis_TdCAT_200_, pHis_TdCAT_295_, pHis_TdCAT_460_, and His_TdCaM1.3) were introduced into the BL21 *E. coli* strain (DE3) (Novagen, Pecs, Hungary). A single selected colony from each construction was grown overnight at 37 °C in LB medium containing 100 µg/mL Kanamycine with shaking at 220 rpm. The culture was next diluted 1:100 into fresh LB-Kanamycine medium and grown to an OD of 0.6 at 600 nm. Protein expression was then induced by 1 mM isopropyl β-D-thiogalactopyranoside (IPTG) overnight at 37 °C. Bacterial cells were harvested by centrifugation at 4500 rpm for 10 min at 4 °C, and the pellets were subsequently washed twice with cold water. Later, the cells were harvested in cold lysis buffer (Tris-HCl 100 mM pH 8; EDTA 1 mM; NaCl 120 mM; 1 mM DTT, 50 mM PMSF, and 0.5% Tween) and sonicated on ice. Afterward, the cells were centrifuged at 9000 rpm for 45 min at 4 °C. The deleted forms were purified from the supernatant, whereas pHis_TdCAT1 was not found in the supernatant. Thus, the recovered inclusion bodies were resuspended and incubated in the lysis buffer overnight at 4 °C with agitation, then centrifuged at 9000 rpm at 4 °C for 10 min. The supernatant was then loaded on Ni-Sepharose column (Bio-Rad, Hercules, CA, USA) pre-equilibrate with binding buffer (Tris-HCl 100 mM pH 8; NaCl 0.5 M; 30 mM imidazole) and gravity eluted. On the other hand, the durum wheat calmodulin protein TdCaM1.3 cloned in frame with GST was expressed and purified as previously described [26]. Protein quantification was performed using the Bradford method [38], and the correct size of recombinant proteins was checked by SDS-PAGE electrophoresis.

### 2.3. CAT Activity Assays

CAT activity was determined according to Feki et al. [39]. In brief, 1 mL of substrate solution made up of 50 mM H_2_O_2_ in a 75 mM phosphate buffer at pH 7.0, 160 µg of proteins were mixed at 25 °C for 1 min, and reaction was stopped by adding 0.2 mL of 1 M HCl. Then, the activity was assayed spectrophotometrically at 240 nm from the rate of H_2_O_2_ decomposition using a specific absorption coefficient at 0.0392 cm^2^μmol^−1^ H_2_O_2_. The CAT activity is expressed as μmol H_2_O_2_ decomposed/mg protein/min. Similar catalase assays were performed lso in the presence or absence of calmodulin and bivalent cations (Mn^2+^, Mg^2+^, Ca^2+^, Fe^2+^, Zn^2+^, Cd^2+^ and Cu^2+^).

### 2.4. Biochemical Characterization of the Catalase TdCAT1

In order to investigate the effect of pH on the catalytic activity, the purified His-TdCAT1 was incubated at room temperature for 10 min in various buffers prior to catalase assays. Catalase activities were measured within a range of pH from 3.0 to 9.0 using 75 mM phosphate buffer. In order to assess the effect of temperature variation, the standard reaction mixtures were pre-incubated at the optimum pH and the indicated temperature (10–80 °C) for 10 min before measuring the catalase activity as indicated above.

### 2.5. GST-Pull Down Assays

Prior to binding, Glutathione Sepharose 4B beads were washed with the appropriate Tris-HCl buffer (Tris-HCl 20 mM; pH 7.4, EDTA 1 mM, DTT 0.5 mM, NaCl 150 mM, 0.5% Triton, PMSF 1 mM) then the same buffer was used to equilibrate those beads. After that, the beads were incubated with 12 μg of GST_TdCaM1.3 or GST alone for 2 h at 4 °C and washed three times to discard the unfixed proteins. Twenty micrograms of the different recombinant forms of His_TdCAT1 proteins were then incubated with the immobilized proteins overnight at 4 °C. After extensive washes, proteins were dissociated from the beads by boiling in Tris-HCl 50 mM, pH 6.8, DTT 1 mM, SDS 2%, glycerol 10%, bromophenol blue 0.1%, then separated by SDS-PAGE (10%). The His-TdCAT1 and the other deleted forms were finally detected by western blot using the anti-Histidine antibody (Sigma, St. Louis, MO, USA) as described by the manufacturer.

### 2.6. Statistical Analysis

Differences between enzymatic reactions in presence of TdCAT1 alone or with CaM and/or cations were analyzed by two-way ANOVA comparison tests with statistical significance set at *p* < 0.05 relative to the control [40].

## 3. Results

### 3.1. Determination of TdCAT1 Activity

**Figure 1:**
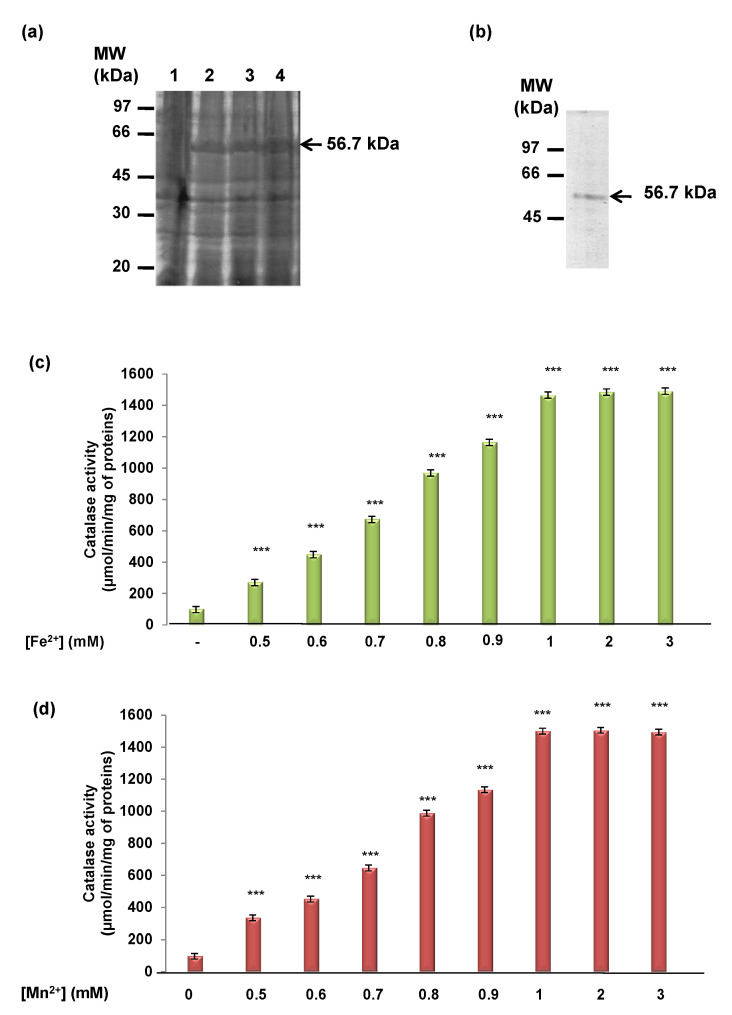
Effects of divalent cations on the catalase activity of TdCAT1. (**a**) Production of durum wheat recombinant His_TdCAT1 protein. Protein extracts from non-induced (lane 1) and IPTG induced (lane 2, 3 and 4) *E. coli* cells expressing pHis_TdCAT1 are presented. The lanes 2, 3 and 4 represent different incubation time in presence of 1 mM IPTG. (**b**) SDS-PAGE analyses of the purified recombinant proteins His_TdCAT1. Positions of the purified proteins are indicated by arrows. The size of protein standards is given in kDa on the left side. (**c**) Stimulatory effects of Fe^2+^ and (**d**) Mn^2+^, on the in vitro catalase activity of the recombinant His-TdCAT1. TdCAT1 activity was assayed with 160 µg of recombinant His_TdCAT1 and 50 mM H_2_O_2_ as a substrate, in the presence of increasing concentrations of Ca^2+^. Values are means of initial rates (µmol/min/mg of proteins) ± S.E from three independent experiments. (***) indicates value significantly different from the control. Statistical significance was assessed by applying the ANOVA test with *p* < 0.005.

After recovery from the inclusion bodies and purification using Ni-Sepharose column chromatography, 160 µg of the recombinant His_TdCAT1 was used for each catalase assay (Figure 1a,b). It is known that the activity of CATs towards their substrate is very low [41]. Thus, the assays were performed using different phosphate buffer concentrations (25, 50, 75, and 100 mM), leading to an optimum phosphate buffer at 75 mM. Moreover, the effect of pH variation (from 3.0 to 9.0) on the decomposition of H_2_O_2_ by TdCAT1 activity was studied. Below pH 5.0, the activity of TdCAT1 was very low (Supplementary Figure S1a), then started to increase by raising the pH to reach a sharp optimum at pH = 7.

For the optimum temperature, we performed a series of CAT activity assays using different temperatures of 10 to 80 °C. Our results show that the catalase activity increases almost proportionally with the temperature rise to reach its maximum at 25 °C and then starts to decrease gradually with higher temperatures (Supplementary Figure S1b). Thus, in this study, the optimal values of buffer concentration, pH, and temperature for TdCAT1 activity were 75 mM, 7, and 25 °C, respectively.

In a second step, we determined the initial reaction rate (Vo) by measuring the enzyme’s kinetics of the purified recombinant proteins His_TdCAT1 during the first min. As it is known that CAT activities can be modulated by bivalent cations [42], and the registered activity in our experimental assays was relatively low (96.27 µmol/min/mg of protein), we investigated whether His_TdCAT1 needs divalent cations to enhance its activity. For this purpose, different enzyme assays were performed with TdCAT1 in the presence of 2 mM of Mn^2+^, Mg^2+^, Ca^2+^, Fe^2+^, Zn^2+^, Cd^2+^, or Cu^2+^. Experimental results showed that the catalytic activity is significantly stimulated in the presence of 2 mM Mn^2+^ and Fe^2+^ and to a lesser extent by Zn^2+^, Cu^2+^, and Ca^2+^ and slightly by Mg^2+^ (Supplementary Figure S1c). In contrast, this activity was not significantly modified by Cd^2+^ (Supplementary Figure S1c). Thus, a dose-response assay was performed with these cations separately, and the results showed that the activity of TdCAT1 is enhanced by increasing Mn^2+^, Ca^2+^, Fe^2+^, Zn^2+^, Cu^2+^, or Mg^2+^ concentrations. In fact, the maximal activity of TdCAT1 (about 16-fold higher than in control conditions) was reached using 1 mM Fe^2+^ (Figure 1c) or Mn^2+^ (Figure 1d). Interestingly, the same result was observed in the presence of Ca^2+^ cations. In fact, the catalase activity of TdCAT1 was stimulated five times more than in the absence of calcium (Supplementary Figure S1d). This stimulation started with 1 mM Ca^2+^ and reached its maximum in the presence of 2 mM Ca^2+^. In the presence of Zn^2+^ and Cu^2+^, the catalytic activity of TdCAT1 also increased with 0.5 mM of both cations (~ about an 8-fold increase) and reached the maximum in the presence of 3 mM (Supplementary Figure S2a,b). In the presence of Mg^2+^, the activity of TdCAT1 also increases in a dose-dependent manner, but to a lesser extent than with Mn^2+^, Fe^2+^, Zn^2+^, Cu^2+^, and Ca^2+^ (Supplementary Figure S2c). Therefore, Mn^2+^ and Fe^2+^ appear to be more efficient than other cations (Mg^2+^, Zn^2+^, Cu^2+^, and Ca^2+^) on the TdCAT1 activity in vitro. Thus, those cations were used to perform the rest of the experiments. Altogether, these results showed that the catalase activity of TdCAT1 can be stimulated by increasing divalent cations in a dose-dependent manner. This stimulation is specific and not artifactual since no activity could be detected when similar assays were performed with a heat-denatured form of His_TdCAT1 incubated at 100 °C for 10 min (data not shown).

On the other hand, the kinetics of the activity of TdCAT1 were investigated in the presence or absence of different cations and at various concentrations (5–70.0 mM) of hydrogen peroxide (in 75 mM phosphate buffer at pH 7.0, 25ar) as a substrate. These data were plotted according to Michaelis–Menten and kinetic parameters, apparent Km and Vmax, calculated from the graphs and using the Michaelis–Menten Equation Calculator (https://www.mdapp.co/michaelis-menten-equation-calculator-431/, accessed on 14 December 2021). The value of apparent Km was 20.97 mM, whereas the Vmax was calculated as 52.8 mM/min. Similar kinetic values were registered in other catalases from plants, such as *Convolvulus arvensis* [43]. It can be observed that divalent cations have induced significant effects on the TdCAT1 activity. By increasing the concentration of SiO_2_ NPs, the kinetic parameters and efficiency of CAT were almost consistent. In fact, the efficacy of the enzyme was 7.1 × 10^7^ and 6.5 × 10^7^ min^−1^mM^−1^ in the absence and presence of 50 μM SiO_2_ NPs, respectively. These data manifest that the CAT efficiency dropped to only 8.5% relative to the native enzyme when the SiO_2_ NPs concentration increased to 50 μM, indicating that SiO_2_ NPs tend to keep the CAT protein in its native state with no significant denaturation.

### 3.2. TdCAT1 Harbors Conserved Ion Binding Motifs Required for Its Activations by Divalent Cations at Different Parts of the Protein

**Figure 2:**
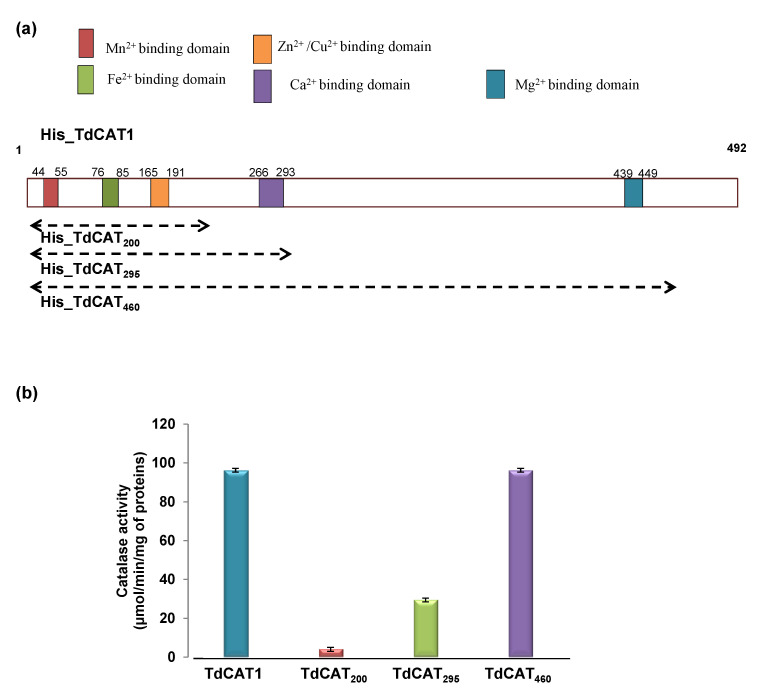
TdCAT1 harbors several putative cations binding domains located at different parts of the protein. (**a**) Schematic presentation of the full length TdCAT1 protein. The position of the different cation binding proteins is indicated. The conserved domains of TdCAT1 including the putative calmodulin Binding Domain CBD and the putative Cation binding domains are presented by boxes with distinct patterns. (**b**) Catalase activity of the different recombinant proteins (160 µg) in presence of 50 mM H_2_O_2_ as a substrate. Values are means of initial rates (µmol/min/mg of proteins) ± S.E from three independent experiments.

The observed stimulatory effects of Fe^2+^, Cu^2+^, Zn^2+^, Mn^2+^, and Mg^2+^ on TdCAT1 activity suggest that durum wheat CAT may harbor cation binding motifs. Thus, we analyzed the structure of TdCAT1 to identify potential cations binding domains. Alignment with well-known Mn^2+^ or Mg^2+^ binding proteins (http://www.uniprot.org (accessed on 14 December 2021)) revealed the presence of putative Mn^2+^ (position 44–55) and Mg^2+^ (position 439–449) binding sites on the N-terminal and the C-terminal region of TdCAT1 sequence respectively (Figure 2a). These domains are highly conserved among CATs proteins from different plant species (Supplementary Figure S3). Moreover, we identified the putative copper/zinc-binding domain, calcium-binding domain, and Iron binding domain in the sequence of TdCAT1. In fact, the analysis of the TdCAT1 amino acids sequence revealed that this protein contains an H-(X)_12_ –H type motif, known as a copper-binding domain, which is localized at 165–177 aa position in the TdCAT1 sequence (HIQENWRILDLFSH, Figure 2a). On the other hand, sequence investigation of zinc-binding domains shows that TdCAT1 contains a domain homolog to the domain identified in protein AN1. This motif binds a single zinc atom (the European Bioinformatic Institute: https://www.ebi.ac.uk/interpro/potm/2007_3/ (accessed on 14 December 2021)) and is localized at the amino acids 163–191 position (Supplementary Figure S4). Moreover, using the Swiss model database (https://swissmodel.expasy.org/interactive/T5XR77/models/ accessed on 14 December 2021), we found a degenerate domain for iron binding (HDV domain) located at 76–85 aa position with conservation of Histidine residue implicated in iron binding. This domain is also well conserved in all studied CATs (data not shown). Finally, using the Supfam databases (http://supfam.org/SUPERFAMILY/cgi-bin/align.cgi accessed on 14 December 2021), a Ca-binding domain called EF-hand (having as consensus the sequence of D-x-[DNS]-{ILVFYW}-[DENSTG]-[DNQGHRK]-{GP}-[LIVMC]-[DENQSTAGC]-x(2)-[DE]) was identified at amino acids position 266–293 of TdCAT1 (Supplementary Figure S5).

To confirm the presence of those putative cation binding domains, we generated three different deleted forms, which are TdCAT_200_ (containing the first 200aa), TdCAT_295_ (containing the first 295aa), and TdCAT_460_ (containing the first 460 aa) (Figure 2a). After the production and purification of those forms, we measured their catalytic activities in the absence of divalent cations. Interestingly, TdCAT_200_ has a very weak basal activity (4.011 µmol/min/mg of protein), whereas TdCAT_295_ has a better catalytic activity (29.41 µmol/min/mg of protein), while TdCAT_460_ and the non-truncated protein TdCAT1 have the same catalytic activity (96.27 µmol/min/mg of protein) (Figure 2b). This result could be explained by the fact that TdCAT1 protein contains one catalase domain (Pfam Id PF00199, 18–399 aa) and catalase-related immune-responsive (Pfam Id PF06628.11, 421–486; data not shown) as revealed by HMMER database. Those results were also shown for some OsCATs demonstrating that the presence of the whole catalase domain is essential for protein activity [41]. Therefore, the entire catalytic domain seems to be important for the catalytic activity of TdCAT1.

**Figure 3:**
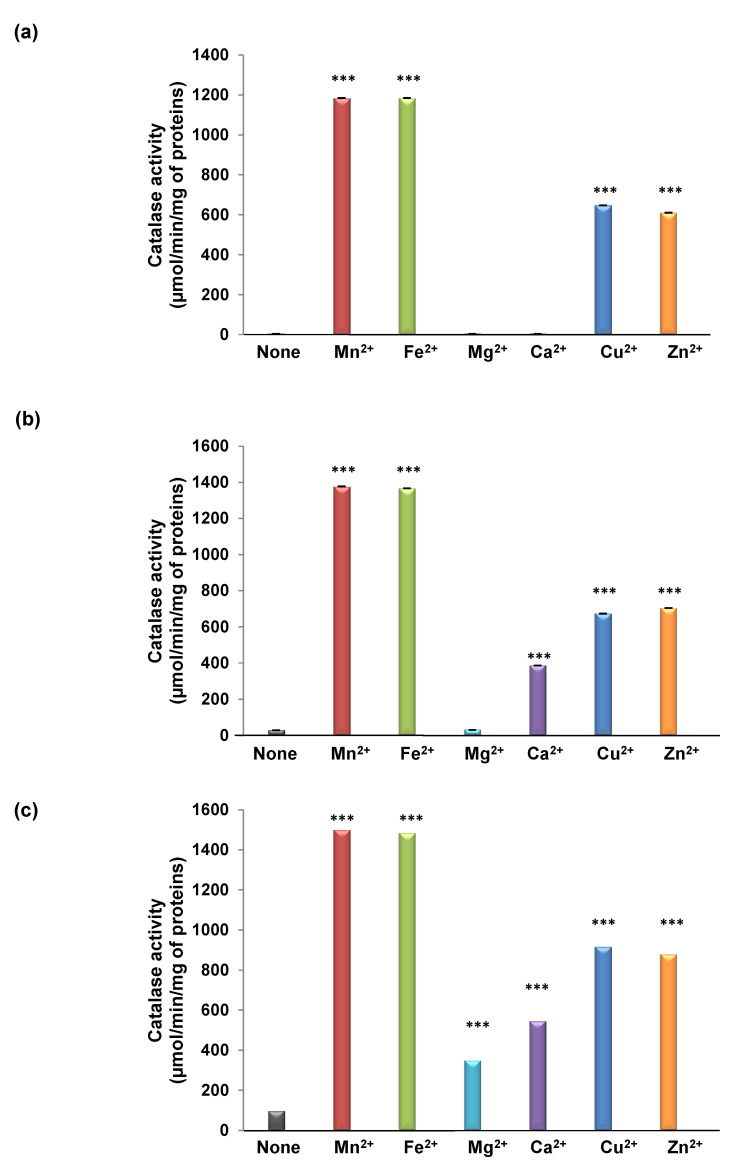
Determination of Catalase activity of the different forms of TdCAT1 with the presence of divalent cations. TdCAT_200_ (**a**), TdCAT_290_ (**b**) and TdCAT_460_ (**c**) were assayed with 160 µg of recombinant protein and 50 mM H_2_O_2_ as a substrate, in presence of 2 mM of Mn^2+^, Fe^2+^, Mg^2+^, Ca^2+^, Cu^2+^ or Zn^2+^. Values are means of initial rates (µmol/min/mg of proteins) ± S.E from three independent experiments. (***) indicates value significantly different from the control. Statistical significance was assessed by applying the ANOVA test with *p* < 0.005.

Following this work, we measured the catalytic activities of those forms in the presence of divalent cations. As expected, TdCAT_200_ activity was stimulated by Mn^2+^, Fe^2+^, Cu^2+^ and Zn^2+^ but not with Ca^2+^ and Mg^2+^ (Figure 3a), confirming that the Ca^2+^ and Mg^2+^ binding domains are not located in the first 200 aa while Mn^2+^, Fe^2+^, Cu^2+^, and Zn^2+^ binding domains could be present in the first 200 aa. Similarly, TdCAT_295_ was stimulated by Mn^2+^, Fe^2+^, Cu^2+^, Ca^2+^ and Zn^2+^ but not with Mg^2+^ (Figure 3b) while TdCAT_460_ was stimulated also by Mg^2+^ (Figure 3c). These results strongly suggest that TdCAT1 can be significantly activated by divalent cations, which may bind to these five different putative cations binding domains distributed along the protein (Figure 2a).

### 3.3. TdCAT1 Harbors a Conserved Calmodulin-Binding Domain

Similar to other plant catalases, TdCAT1 protein contains a putative CaMBD located at the C-terminal region of TdCAT1 positioned from residues 413 to 453 (Supplementary Figure S6a) [32]. Protein sequence analysis by the calmodulin target database (http://calcium.uhnres.utoronto.ca (accessed on 14 December 2021)) server revealed that this domain is rather located at 459-482aa (Supplementary Figure S6b). Moreover, in silico analyses using interpret server (https://www.ebi.ac.uk/interpro/entry/IPR000048 (accessed on 14 December 2021)) reveal a putative interaction between TdCAT1 and CaMs. The construction of the helical wheel model showed that this CaMBD contains basic and hydrophobic amino acids with the conserved tryptophane residue (53.54%, Supplementary Figure S6c,d) and segregated into the opposite side of the helix, which has been named the basic amphiphilic_α helix (Baa) motif (data not shown).

### 3.4. TdCAT1 Interacts In Vitro with TdCaM1.3

**Figure 4:**
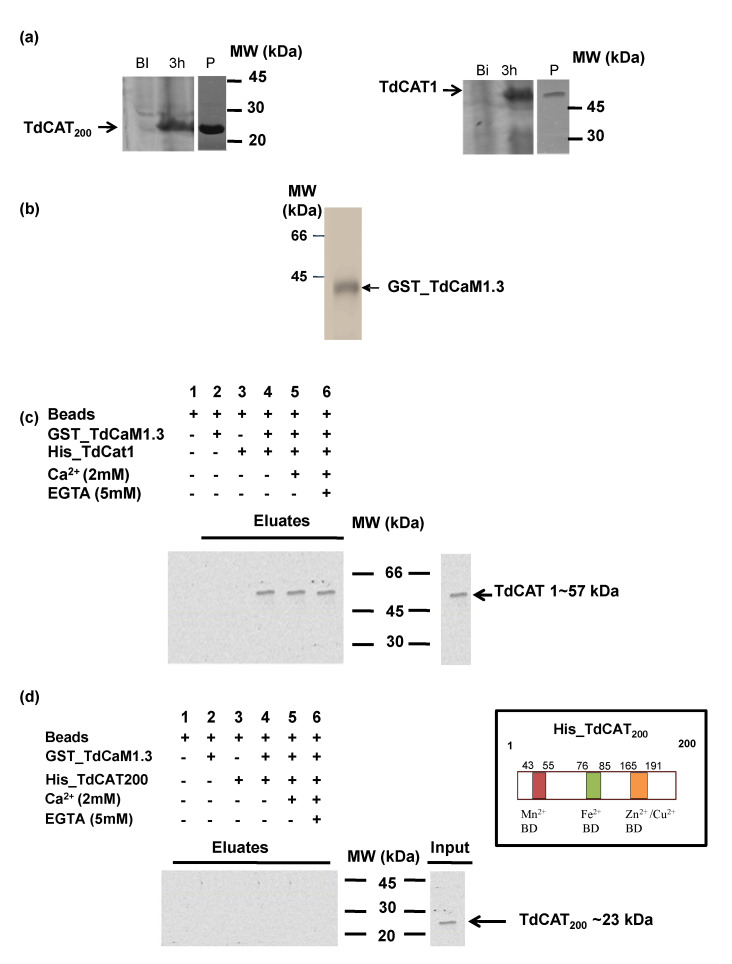
TdCaM1.3 interacts with type 1 Catalase proteins (TdCAT1). (**a**) Purification of recombinant proteins TdCAT1 and the deleted form TdCAT_200_. (**b**) Purification of recombinant GST-TdCaM1.3 proteins. (**c**) Physical interaction of TdCaM1.3 and TdCAT1 in vitro was verified by GST pull-down assay. GST-TdCaM1.3 was incubated in binding buffer containing glutathione-agarose beads with or without His_TdCAT1 and agarose beads were washed for five times and eluted. Lysis of *E. coli (BL21)* (Input) and eluted proteins (Pull-down) from beads was immunoblotted using anti-His antibodies. (**d**) The deleted TdCAT_200_ form does not interact with GST-TdCaM1.3 by GST pull down assays as it was detected in the eluates.

To check the regulation of TdCAT1 by CaMs, we investigated the functional properties of its CaMBD domain. For this purpose, in vitro, GST-pull down assay was performed using as a bait the previously described recombinant CaM from durum wheat, GST-TdCaM1.3 as [26] and His_TdCAT1 (Figure 4a,b). The purified His_TdCAT1 was mixed with the Nickel beads bound GST_TdCaM1.3, and the interaction between the two proteins was investigated by immune blotting the membrane using the anti-Histidine antibody. As shown in Figure 4c, the His_TdCAT1 was pulled down by the GST_TdCaM1.3 (lane 3) but not with beads alone (lane 1), while the GST_TdCaM1.3 was not detected using the anti-histidine tag antibodies (lane 2). This interaction appears to be Ca^2+^ independent since the signal corresponding to TdCAT1 is detected when the GST_TdCaM1.3 was supplemented with 2 mM Ca^2+^ (Figure 4c, lane 4) and or in the presence of the chelating agent EGTA (5 mM) (Figure 4c, lane 5). Thus, TdCAT1 harbors a conserved calmodulin-binding domain and interacts with CaMs in a calcium-independent manner, contrary to other identified catalases isolated from Arabidopsis and potato. In order to confirm this result, the truncated form His-TdCAT_200_ (containing no putative CaMBD) was used for similar pull-down assays. As expected, His_TdCAT_200_ was not pulled down by GST_TdCaM1.3 (Figure 4d), confirming that TdCAT1 interaction to CaM is specific and requires the conserved the CaMBD motif.

### 3.5. Effects of TdCaM1.3 on TdCAT1 Activity

**Figure 5:**
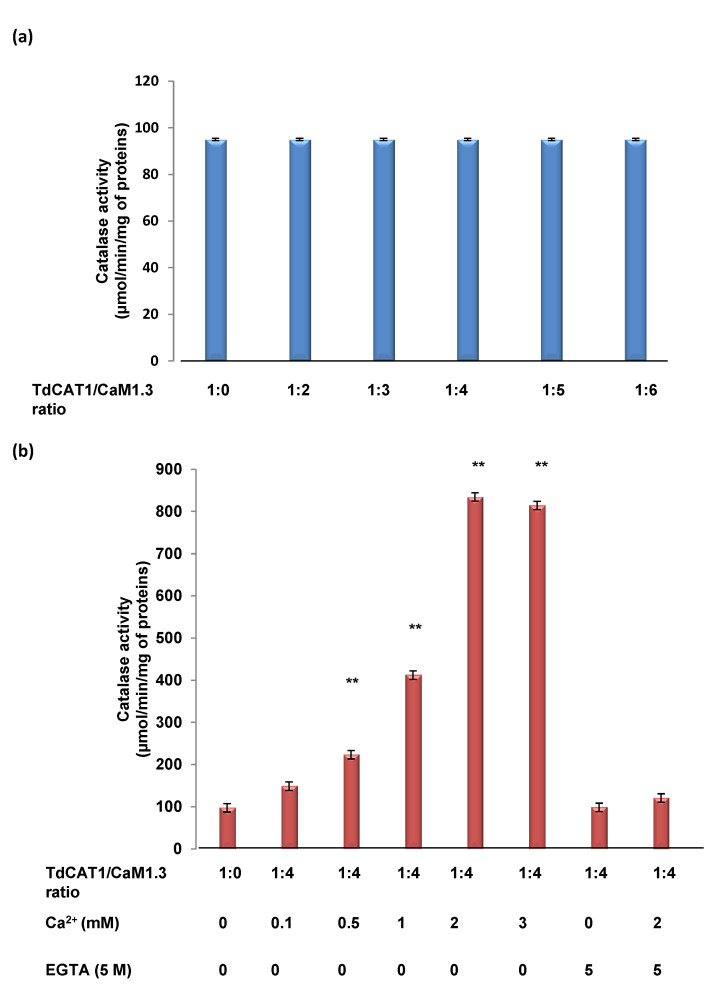
Effects of the TdCaM1.3/Ca^2+^ complex on TdCAT1 activity. (**a**) TdCaM1.3 alone has no effect on catalase activity of TdCAT1. (**b**) Stimulatory effect of the TdCaM1.3/Ca^2+^ complex in presence of increasing concentrations of Ca^2+^ from 0 to 3 mM and EGTA (5 mM) as indicated. Catalase assays were performed in the same buffer conditions as mentioned in presence of TdCAT1/tdCaM1.3 ratio molar 1:4. All data are mean values ± S.E of initial rate from three independent assays. (**) indicates value significantly different from the control. Statistical significance was assessed by applying the ANOVA test with *p* < 0.005.

**Figure 6:**
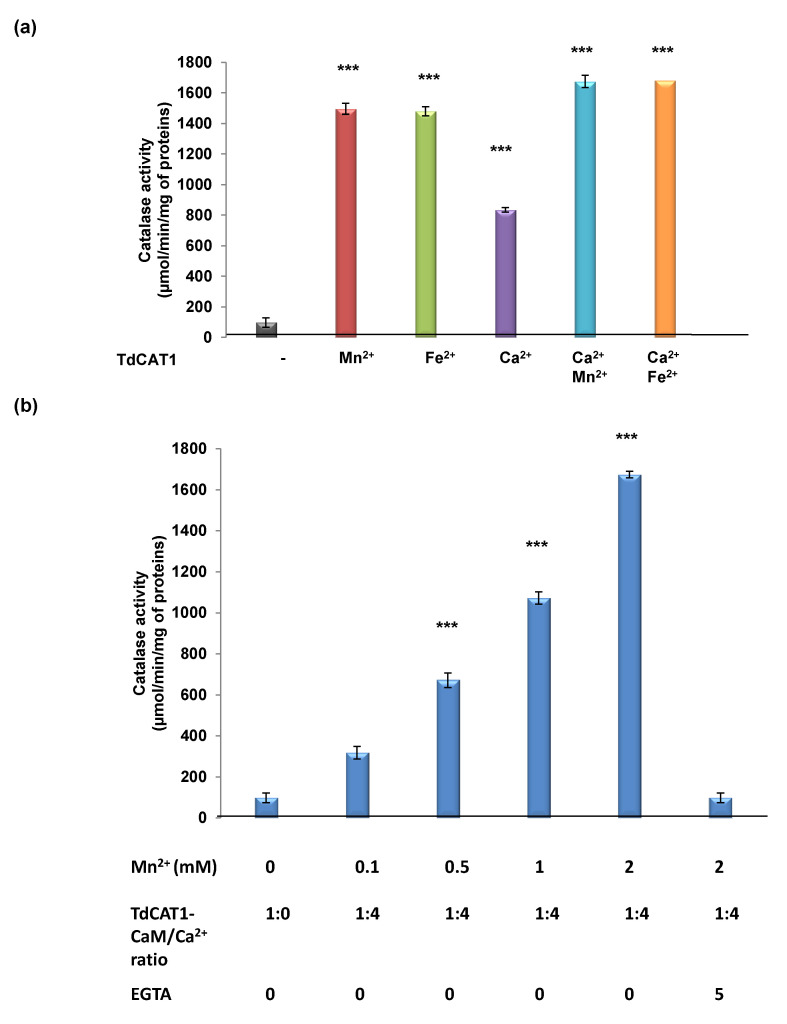
Effects of Mn^2+^ cations and CaM/Ca^2+^ complex on TdCAT1 activity. (**a**) Ca^2+^ cations further enhance catalase activity of TdCAT1 in presence of Mn^2+^ and Fe^2+^. (**b**) Stimulatory effect of TdCaM1.3/Ca^2+^ complex on His_TdCAT1 activity in the same buffer conditions using increasing concentrations of Mn^2+^ and in presence of TdCAT1/TdCaM1.3 ratio molar of 1:4. All data are mean values ± S.E of initial rate from three independent assays. (***) indicates value significantly different from the control. Statistical significance was assessed by applying the ANOVA test with *p* < 0.005.

It has been demonstrated that CaM proteins interact with various target proteins and modulate their activities [22,26,27,44]. Consequently, we investigated the effect of wheat calmodulin binding on the catalase activity of TdCAT1 in vitro. As shown in Figure 5a, in the absence of Ca^2+^, TdCaM1.3 alone did not modify TdCAT1 activity. When the calcium is added to the reaction medium, both His-TdCaM1.3 (Figure 5b) and GST-TdCaM1.3 (data not shown) stimulated the catalytic activity of TdCAT1 with the same fold, and it reached the maximum (8 times increase in the Vo) in the presence of 2mM of Ca^2+^. Moreover, the addition of EGTA decreases the activity of TdCAT1 in the presence of TdCaM1.3/Ca^2+^ (Figure 5b) to its basal level, indicating that Ca^2+^ is necessary for the activation of TdCAT1 by TdCaM1.3. As Mn^2+^ and Fe^2+^ were shown to enhance the TdCAT1 catalytic activity (Figure 2c), we also evaluated the effects of TdCaM1.3/Ca^2+^ on the TdCAT1 activity in the presence of those cations. Remarkably, in a buffer containing 2 mM of Mn^2+^ or Fe^2+^, the addition of Ca^2+^ slightly increases the catalase activity of TdCAT1 (Figure 6a). In the presence of the TdCaM1.3/Ca^2+^ complex, the addition of Mn^2+^ further stimulates the activity of TdCAT1 (Figure 6b). This stimulatory effect of TdCaM1.3 occurs albeit with lower efficiency even with concentrations of Mn^2+^ and Ca^2+^ as low as 0.5 mM (Figure 6b); the increase reached its maximum at 2 mM. This increase is calcium-dependent because the addition of EGTA is sufficient to return the TdCAT1 activity to its initial level (Figure 6b). Finally, to confirm the observed stimulatory effects of TdCaMs/Ca^2+^ on TdCAT1 activity, we performed a new series of catalase assays using the truncated form His_TdCAT_200_. As expected, the activity of His_TdCAT_200_ remains unchanged in the presence of TdCaMs/Ca^2+^. There is neither a negative (in the absence of Ca^2+^; Supplementary Figure S7a) nor a positive effect (in the presence of TdCAT1/TdCaM1.3 ratio molar of 1:4 and Ca^2+^; Supplementary Figure S7b) of TdCaM1.3 on the catalytic activity of this truncated TdCAT_200_ protein mainly in the presence of increasing quantities of CaMs in the medium (data not shown). The same effect is observed in the presence of Mn^2+^ and Fe^2+^ cations (Supplementary Figure S7c). Altogether, these data confirm that the catalytic activity of TdCAT1 can be specifically activated by CaM/Ca^2+^ in the presence of Mn^2+^ and Fe^2+^.

## 4. Discussion

Reactive oxygen species are toxic byproducts of metabolism. Those unavoidable components act as signaling molecules under normal and stressful conditions [45]. In plants, ROS are generated from the reduction of atmospheric oxygen. ROS are also generated in chloroplasts [46] and mitochondria [47] via the electron transport chains and in peroxisomes during photorespiration [48]. In the apoplast, ROSs could also be produced via the plasma membrane-localized NADPH oxidase, oxalate oxidase, or by the degradation of spermidine by polyamine oxidase [49]. At low cellular concentrations, ROS stimulates the expression of a large variety of stress-responsive genes. At higher levels, ROS may cause severe damage to proteins, lipids, and nucleic acids, thus causing cell death [50]. Only four ROS are more abundant and stable (known as singlet oxygen (_1_O^2^), hydroxyl radical (OH^·^), hydrogen peroxide (H_2_O_2_), and superoxide (O_2_^−^)). They differ in their reactivity and ability to be transported across membranes. They also differ in their stability. Hydrogen peroxide (H_2_O_2_) is the most stable ROS [51]. ROS signaling can be propagated for long distances (from cell to cell) in a process called ROS waves [52], mediated by different cell components such as calcium (Ca^2+^) channels [53]. ROS are involved in different developmental processes as well as plant stress responses [49]. Enzymatic antioxidant capacity contributes to plant survival in adverse conditions. Ascorbate peroxidases (APXs), monodehydroascorbate reductases (MDHARs), catalases (CATs), superoxide dismutases (SODs), and glutathione reductases (GRs) are among the main antioxidant enzyme classes. Among those enzymes, CATs are the major enzymes implicated in the detoxification of H_2_O_2_ into H_2_O and O_2_ [54].

Plant catalases have been studied in many species such as *Arabidopsis*, sweet potato, pumpkin, and wheat [30,32]. These proteins are involved in the detoxification of H_2_O_2_ into water and oxygen in all aerobic organisms. They are activated under developmental processes and in response to environmental stimuli [7]. In this work, the in vitro catalase activity of durum wheat TdCAT1 was investigated using the recombinant form His_TdCAT1. Characterization of the His_TdCAT1 activity revealed that this oxydo-reductase had no activity in high acidic mediums such as CATs from rice (OsCAT-A, OsCAT-B, and OsCAT-C), but it exhibited maximum activity at pH 7 (Supplementary Figure S1a). Such results were previously shown for *Oryza sativa* OsCAT-C [55] and *Triticum aestivum* TaCAT1 [56]. Those enzymes exhibit optimal activity at 25 °C before starting their decrease gradually with higher temperatures (Supplementary Figure S1b), as shown for OsCAT-B [55]. TdCAT1 activity was stimulated in presence of different divalent cations (Ca^2+^, Mn^2+^, Mg^2+^, Fe^2+^, Cu^2+^, Zn^2+^) but not in presence of Cd^2+^ (Supplementary Figure S1c). Moreover, the alignment of TdCAT1 with well-known Mn^2+^ and Fe^2+^ binding proteins (using http://www.uniprot.org, accessed on 14 December 2021) confirmed the presence of putative Mn^2+^/Fe^2+^ binding domains in the N-terminal region of the TdCAT1 sequence. Interestingly, the catalase activity of TdCAT1 was stimulated up to 16-fold in the presence of Mn^2+^ and Fe^2+^ cations (Supplementary Figure S1c). A similar stimulatory effect was also observed in the presence of Ca^2+^ cations (about 8-fold induction; Supplementary Figure S1). The positive effects of these divalent cations corroborate with the presence of Mn^2+^, Fe^2+^, Cu^2+^/Zn^2+^, Ca^2+^ and Mg^2+^ binding domains in the TdCAT1 structure (Figure 2a). Mn^2+^ and Mg^2+^ binding domains are conserved in all studied catalase proteins (Supplementary Figure S3) and shared the same characteristics with other known cations binding domains (Supplementary Figure S4). Both metal cations are frequently bound by Aspartate (D) and Glutamate (E) residues situated in “β strand—random coil—β strand” and “β strand—random coil—α helix” structural motifs [57]. In association with Asp and Glu residues, Histidine residues are considered a major binder of Mn^2+^cations [58].

Putative Ca^2+^ binding domains were also identified in the sequence of TdCAT1. Those domains have a degenerated EF-hand motif D-x-[DNS]-{ILVFYW}-[DENSTG]-[DNQGHRK]-{GP}-[LIVMC]-[DENQSTAGC]-x(2)-[DE].

Potential copper-interacting motifs were predicted and scored in *Arabidopsis* via copper-immobilized metal affinity chromatography (Cu-IMAC) [58]. Six candidate motifs, H-(X)_5_-H, H-(X)_7_-H, H-(X)_12_-H, H-(X)_6_-M, M-(X)_7_-H, and H-(X)_3_-C, are present in Cu-IMAC- isolated proteins with higher frequency than in the whole Arabidopsis proteome. Here, the sequence analysis of TdCAT1 showed that the protein contains an H-(X)_12_-H-binding motif type, which may explain its activation by Cu^2+^.

Therefore, as far as we know, we demonstrate here for the first time that plant catalases harbors cation-binding domains which can be behind the observed activation of TdCaM1 by Mn^2+^, Fe^2+^, Mg^2+^, Cu^2+^, Zn^2+^, and Ca^2+^. An additional observation came from the deleted His-TdCAT_200_ and His-TdCAT_295_ forms (where the C-terminal part, including the catalase domain and the catalase-related immune-responsive, were deleted). Those forms showed a decrease in catalase activity compared to full length and His-TdCAT_460_ forms (Figure 2b). Interestingly, His-TdCAT_200_ was stimulated in the presence of Mn^2+^, Fe^2+^, Zn^2+^, and Cu^2+^ but not with Ca^2+^ and Mg^2+^, confirming that Mn^2+^, Fe^2+^, Zn^2+^, and Cu^2+^ binding domains are located in the first 200 aa of TdCAT1 (Figure 3a). Moreover, His-TdCAT_295_ was also stimulated in the presence of calcium (Figure 3b) whereas His-TdCAT_460_ was stimulated in the presence of all cations (Figure 3c). Fine mapping of this region is needed to locate more precisely the different divalent cation-biding motifs.

In a second step, we identified a putative calmodulin-binding domain in the TdCAT1 sequence located at its C-terminal part (459–482aa; Supplementary Figure S6). It was reported that this domain is essential for calmodulin binding and activation of some plant CATs in a calcium-dependent manner [30]. Calmodulins (CaMs) are ubiquitous small proteins containing only four motifs called Ef-Hands, a typical feature of Ca^2+^ binding proteins [30]. CaMs mediate the primary intracellular Ca^2+^ signaling pathways, and elevation in Ca^2+^ concentration in the nucleus or in the cytosol induces the formation of Ca^2+^/CaM complexes which interact with an important number of targets such as ion transporters, protein kinases, pathogen-related proteins, transcription factors, and protein phosphatases and regulate cellular functions [22,24,26,27]. In plants, the biological significance of the interaction between CAT proteins and CaM/Ca^2+^ complexes remain poorly investigated. In this study, we demonstrated that the wheat catalase TdCaM1.3 interacts with TdCAT1 in a Ca^2+^-independent manner (Figure 4). The binding of CaM/Ca^2+^ complex to TdCAT1 results in the activation of its catalytic activity in vitro (Figure 5). Moreover, the addition of Mn^2+^ cations to the CaM/Ca^2+^ complex slightly increases the catalase activity of TdCAT1 (Figure 6). The positive Mn^2+^-mediated effect of CaM/Ca^2+^ requires a direct interaction between TdCAT1 and Mn^2+^ via the C-terminal part of the protein as the deleted form His-TdCAT_200_ (Supplementary Figure S7). Recently, the positive role of Mn^2+^ cations in stimulating the effects of the CaM/Ca^2+^ effect has been described. In fact, in durum wheat, mitogen-activated protein kinase phosphatase 1 (TMKP1) was described to be inhibited by CaM/Ca^2+^ complex in a dose-dependent manner. However, the addition of Mn^2+^ suppresses this negative effect, and the CaM/Ca^2+^/Mn^2+^ complex stimulates the phosphatase activity of the protein two-fold [27]. Moreover, TdPR-1 protein activity was also stimulated in the presence of the CaM/Ca^2+^/Mn^2+^ complex. In absence of Mn^2+^ cations CaM/Ca^2+^ complex had no effect on the PR-1 protein activity [26]. Altogether, these results suggest that Mn^2+^ cations act as a cofactor in the activation of TdCAT1 by the TdCaM1.3/Ca^2+^ complex.

## 5. Conclusions

The data obtained in this study regarding TdCAT1 provide evidence for a novel regulatory mechanism where divalent cations can modulate the catalase activity in vitro. The catalytic activity of TdCAT1 is stimulated by Fe^2+^ and Mn^2+^ cations and, to a lesser extent, in the presence of Zn^2+^, Cu^2+^, and Mg^2+^ in a dose-dependent manner. The cations binding domains are conserved in many catalases, which suggests a conserved mode of regulation for these proteins. Catalytic TdCAT1 activity was also shown to be stimulated by CaM/Ca^2+^ complex. Therefore, the wheat catalase seems to be stimulated by the synergistic action of Mn^2+^/CaM/Ca^2+^. Further experiments are conducted to reveal the role of those domains in planta by generating transgenic plants overexpressing different forms of catalase gene with no cations binding domains. Moreover, as CaM/Ca^2+^ complex enhances the catalytic activity of TdCAT1, it would be interesting to generate transgenic plants overexpressing catalase/calmodulin proteins to study the role of this complex in alleviating plant responses to surrounding stresses. Such functional studies should help to understand the significance of these stimulatory effects on plant catalases in the control of plant responses to abiotic and/or biotic stresses.

## Data Availability

Data are contained within the article or supplementary material.

## Supplementary Materials

The following supporting information can be downloaded at: https://www.mdpi.com/article/10.3390/antiox11081483/s1, Figure S1: Functional characterization of TdCAT1.; Figure S2: Stimulatory effects of Zn^2+^, Cu^2+^ and Mg^2+^ on the in vitro catalase activity of the recombinant His-TdCAT1.; Figure S3: Sequence alignment of Mn^2+^ and Mg^2+^ binding domains from different plant catalase.; Figure S4: Sequence alignment of Zn/Cu binding domain from different plant catalase.; Figure S5: Sequence alignment of Ca^2+^ binding domain from different plant catalase.; Figure S6: TdCAT1 harbors a conserved CaMBD located at the C-terminal part of the protein.; Figure S7: The activity of His_TdCAT1_200_ is not affected by TdCaM1.3/Ca^2+^ complex.; Table S1: List of primers used in PCR amplification of TdCAT1 and its truncated forms.
