# Supplementary material for: The Activity of the Durum Wheat (Triticum durum L.) Catalase 1 (TdCAT1) Is Modulated by Calmodulin
Source: Antioxidants (Basel). 2022 Jul 29;11(8):1483. doi: 10.3390/antiox11081483 (PMC9404813; doi:10.3390/antiox11081483)
Supplement: Supplementary file 1 [file antioxidants-11-01483-s001.zip › antioxidants-1763377-supplementary.pdf]

Supplemental Table S1. List of primers used in PCR amplification of TdCAT1 and its truncated forms. The forward primer CF was used for the amplification of all forms with the use of reverse primers corresponding of each one.

Amplified fragments	Nucleotide sequences	Primers names
	TCGAATTCATGGACCCCTACAAGTA	CF
TdCAT1	ATCTCGAGTTACATGCTCGGCTTGG	CR1
TdCAT$_{200}$	ATCTCGAGGGCGGTAGTCGGCGGG	CR2
TdCAT$_{295}$	ATCTCGAGCCTCGGGCCACGTCTTG	CR3
TdCAT$_{460}$	ATCTCGAGGGTGAGGCGGGGGTCC	CR4

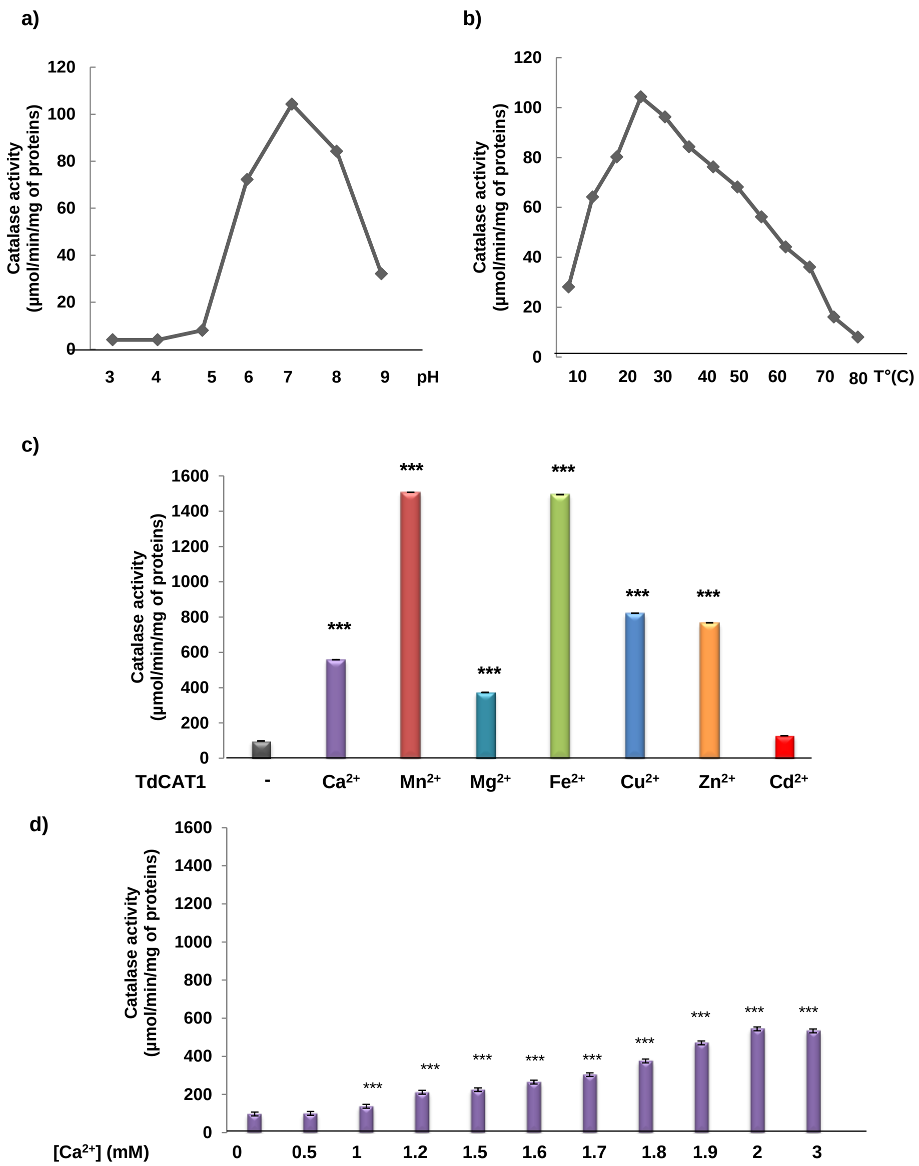

Supplemental Figure S1. Functional characterization of TdCAT1. (a) Effect of pH on the catalytic activity of TdCAT1. pH stability of the catalytic activity of TdCAT1 was studied in different pH medium ranging from 3-9 with a maximum activity at pH 7. **(b)** Effect of temperature on the catalytic activity of TdCAT1. The catalytic activity was studied in presence of different temperature with 5°C difference graduation and maximum activity at 25°C. **(c)** Stimulatory effects of divalent cations on the *in vitro* phosphatase activity of the recombinant His_TdCAT1. The catalytic activity is stimulated in the presence of 2 mM Mn^{2+}, Fe^{2+}, Zn^{2+}, Cu^{2+}, Mg^{2+} and Ca^{2+} but not in presence of Cd^{2+}. **(d)** stimulatory effect of Ca^{2+} cations on the catalase activity of TdCAT1. TdCAT1 activity was assayed with 160 µg of recombinant Protein after incubation for 1 min in presence of 50mM H_2O_2 as a substrate in a 75 mM phosphate buffer at pH 7.0 and 25°C and the absorbance was measured at 240 nm. H_2O_2 decomposition was measured by measuring the decrease of H_2O_2 absorbance and was calculated as µmol H_2O_2 decomposed/mg protein/min. Values are means of initial rates (µmol/min/mg of proteins) ± S.E from at least three independent experiments with identical results.

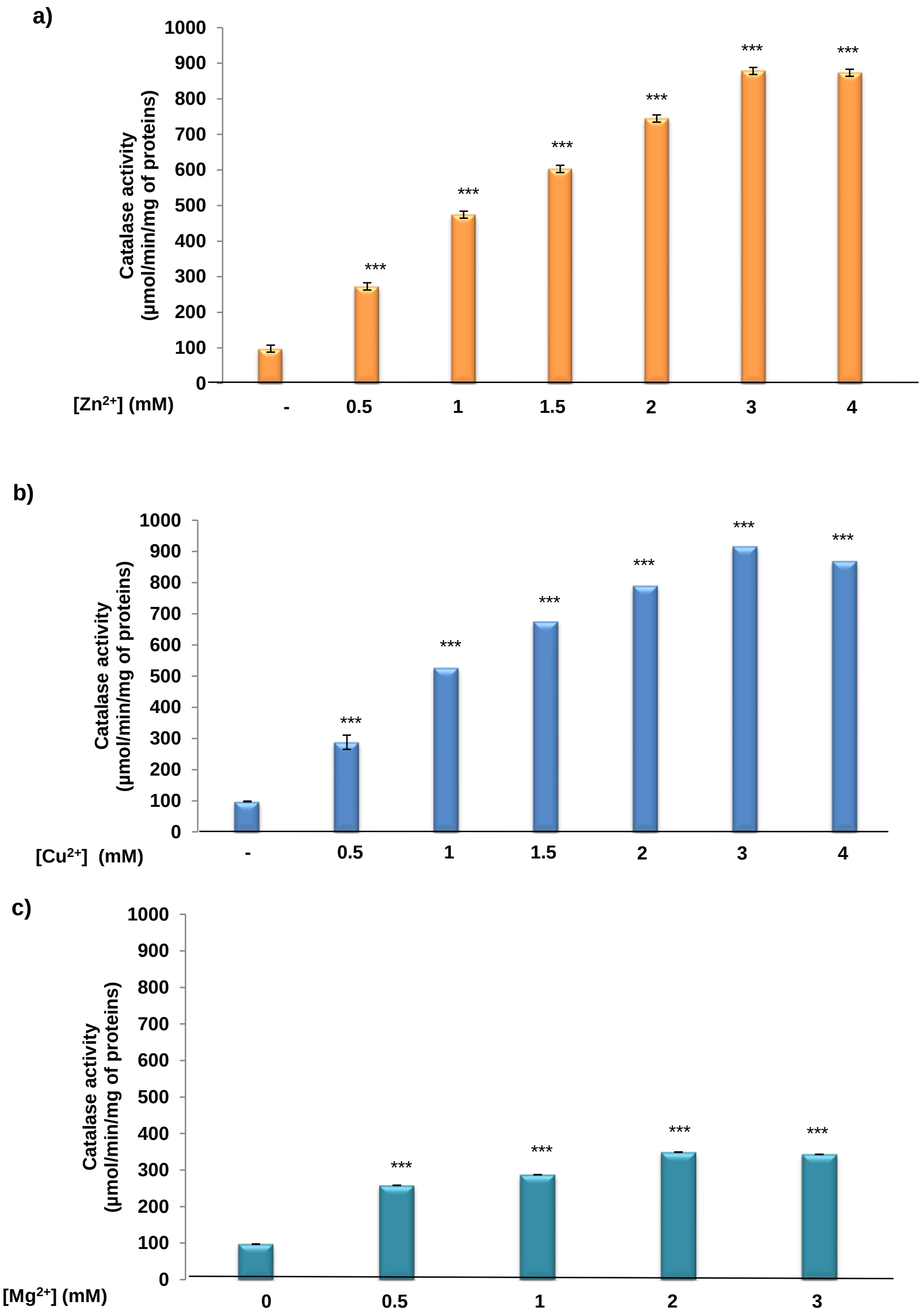

Supplemental Figure S2. Stimulatory effects of Zn^{2+}, Cu^{2+} and Mg^{2+} on the *in vitro* catalase activity of the recombinant His-TdCAT1. TdCAT1 activity was assayed with 160 µg of recombinant His_TdCAT1 and 50 mM H_2O_2 as a substrate, in the presence of increasing concentrations of Zn^{2+} **(a)**, Cu^{2+} **(b)**, and Mg^{2+} **(c)**. Values are means of initial rates (µmol/min/mg of proteins) ± S.E from three independent experiments. (***) indicates values significantly different from the control (basal catalase activity of TdCAT1). Statistical significance was assessed by applying the ANOVA test with <0.005.

a)

Mn^{2+} binding Domain

```
SlCat3      DYHLLEKIANFD   12
OsCat       DYHLVEKLANFD   12
ZmCat2      DYHLVEKLANFD   12
AtCat1      DYHLVEKLANFD   12
NtCat1      DYHLVEKLANFD   12
NbCat       DYHLVEKLANFD   12
GhCat1      DYHLVEKLANFD   12
StCat2      DYHLVEKLANFD   12
AtCat2      DYHLVEKLANFD   12
TdCat1      DYHLVEKIADFD   12
AetCaT1     DYHLVEKIADFD   12
BdCat1      DYHLVEKIANFD   12
AtCat3      DYHLIEKVANFT   12
SpCat1      DYHLIEKIQNFT   12
            ****:**:  :*
```

b)

Mg^{2+} binding Domain

```
AtCat1      TPERQERFIQR    11
AtCat2      TPERQERFIQR    11
NtCat1      TPDRQERFIRR    11
NbCat       TPDRQERFIRR    11
StCat2      TPDRQERFIRR    11
TdCat1      DPARQERFINR    11
BdCat1      DPARQERFINR    11
AetCaT1     DPARQERFINR    11
ZmCat2      DPARQERFITR    11
OsCat       DPARQDRFIKR    11
SlCat3      APDRQERFLCR    11
GhCat1      AADRQERFICR    11
AtCat3      APDRQDRFVKR    11
SpCat1      APDRQDRFINR    11
               **:**: *
```

Supplemental Figure S3. Sequence alignment of Mn^{2+} and Mg^{2+} binding domains from different plant catalase. *Arabidopsis thaliana* [CAT1 (accession no. X94447), CAT2 (accession no. X64271) and CAT3 (accession no. U43147)], rice *Oryza sativa* (OsCAT A; accession no.XP_015629749.1), *Zea mays* (ZmCAT1; accession no. PWZ57146.1), *Nicotiana tabacum* (NtCAT; accession no. U93244), *Nicotiana bentamiana* (NbCAT; accession no. EU998969), *Gossypium hirsutum* (GhCAT1; accession no. XP_016687939.1), *Solanum tuberosum* (StCAT2, accession no. KAH0685113.1), *Triticum turgidium ssp durum* (TdCAT1; Accession no. KP696753), *Aeogilops tauchii* (AetCAT1; accession no. XP_020164896.1), *Brachypodium dyctiosum* (BdCAT1, accession no. XP_003558892.1) and *Sorghum bicolor* (SbCAT1, accession no. XP_021304262.1).

TdCAT1	**H**	I	Q	E	N	W	R	I	L	D	L	F	S	**H**	H	P	E	S	L	H	M	F	T	F	L	F	D
BdCAT1	**H**	I	Q	E	N	W	R	I	L	D	F	F	S	**H**	H	P	E	S	L	H	M	F	T	F	L	F	D
OsCAT	**H**	V	Q	E	N	W	R	I	L	D	F	L	S	**H**	H	P	E	S	L	H	M	F	T	F	L	F	D
ZmCAT2	**H**	I	Q	D	N	W	R	I	L	D	F	F	S	**H**	H	P	E	S	L	H	M	F	S	F	L	F	D
AtCAT1	**H**	I	Q	E	N	W	R	I	L	D	F	F	S	**H**	H	P	E	S	L	N	M	F	T	F	L	F	D
AtCAT2	**H**	I	Q	E	N	W	R	I	L	D	F	F	S	**H**	H	P	E	S	L	N	M	F	T	F	L	F	D
AtCAT3	**N**	I	Q	E	Y	W	R	I	L	D	Y	M	S	**H**	L	P	E	S	L	L	T	W	C	W	M	F	D
NtCAT1	**H**	I	Q	E	N	W	R	V	L	D	F	F	S	**H**	V	P	E	S	L	H	M	F	T	F	L	F	D
NbCAT1	**H**	I	Q	E	N	W	R	V	L	D	F	F	S	**H**	V	P	E	S	L	H	M	F	T	F	L	F	D
SlCAT3	**H**	I	Q	D	N	W	R	I	L	D	F	F	S	**H**	H	P	E	S	L	H	M	F	S	F	L	F	D
StCAT2	**H**	I	Q	E	N	W	R	I	L	D	F	F	S	**H**	H	P	E	S	L	N	M	F	T	F	L	F	D
GhCAT	**H**	I	Q	E	N	W	R	I	L	D	F	F	S	**H**	H	P	E	S	L	N	M	F	T	F	L	F	D
SpCAT1	**H**	I	Q	E	N	W	R	I	L	D	Y	L	S	**H**	L	P	E	S	L	N	T	F	A	W	F	Y	D

Supplemental Figure S4. **Sequence alignment of Zn/Cu binding domain from different plant catalase**. *Arabidopsis thaliana* [CAT1 (accession no. X94447), CAT2 (accession no. X64271) and CAT3 (accession no. U43147)], rice *Oryza sativa* (OsCAT A; accession no. XP_015629749.1), *Zea mays* (ZmCAT1; accession no. PWZ57146.1), *Nicotiana tabacum* (NtCAT; accession no. U93244), *Nicotiana bentamiana* (NbCAT; accession no. EU998969), *Gossypium hirsutum* (GhCAT1; accession no. XP_016687939.1), *Solanum tuberosum* (StCAT2, accession no. KAH0685113.1), *Triticum turgidium ssp durum* (TdCAT1; Accession no. KP696753), *Aeogilops tauchii* (AetCAT1; accession no. XP_020164896.1), *Brachypodium dyctiosum* (BdCAT1, accession no. XP_003558892.1) and *Sorghum bicolor* (SbCAT1, accession no. XP_021304262.1).

	E	n	-	-	n	n	-	-	n	x	-	y	-	S	G	-	-y	-	-	-	-z
	**E**									**D**	X	D NS	IL VF Y W	**DE NS TG**	D N Q G H RK	G P	LI V M C	**DE N Q ST A G C**	X	X	**DE**
TdCAT1	**E**	W	T	F	Y	I	Q	T	I	**D**	P	D	H	**E**	E	R	F	**D**	-	F	**D**
BdCAT1	**E**	W	K	F	Y	I	Q	T	I	**D**	P	D	H	**E**	G	R	F	**D**	-	F	**D**
OsCAT	**E**	W	K	L	F	I	Q	T	I	**D**	P	D	H	**E**	D	R	F	**D**	-	F	**D**
ZmCAT2	**E**	W	T	L	Y	I	Q	T	M	**D**	P	E	M	**E**	D	R	L	**D**	D	L	**D**
AtCAT1	**E**	W	K	L	F	I	Q	I	I	**D**	P	A	D	**E**	D	K	F	**D**	-	F	**D**
AtCAT2	**E**	W	K	L	F	I	Q	I	I	**D**	P	A	D	**E**	D	K	F	**D**	-	F	**D**
AtCAT3	**E**	W	K	L	F	I	Q	T	M	**D**	P	A	D	**E**	D	K	F	**D**	-	F	**D**
NtCAT1	**E**	W	K	L	F	I	Q	T	M	**D**	P	D	H	**E**	D	R	F	**D**	-	F	**D**
NbCAT1	**E**	W	K	L	F	I	Q	T	M	**D**	P	D	H	**E**	D	R	F	**D**	-	F	**D**
SlCAT3	**E**	W	K	L	F	I	Q	T	I	**D**	P	D	H	**E**	D	R	F	**D**	-	F	**D**
StCAT2	**E**	W	K	L	F	I	Q	I	M	**D**	P	D	H	**E**	D	K	F	**D**	-	F	**D**
GhCAT1	**E**	W	K	L	F	I	Q	T	I	**D**	P	D	H	**E**	D	K	F	**D**	-	F	**D**
SpCAT1	**E**	W	K	L	Y	I	Q	V	M	**D**	P	D	H	**E**	D	R	F	**D**	-	F	**D**

Supplemental Figure S5. Sequence alignment of Ca^{2+} binding domain from different plant catalase. *Arabidopsis thaliana* [CAT1 (accession no. X94447), CAT2 (accession no. X64271) and CAT3 (accession no. U43147)], rice *Oryza sativa* (OsCAT A; accession no. XP_015629749.1), *Zea mays* (ZmCAT1; accession no. PWZ57146.1), *Nicotiana tabacum* (NtCAT, accession no. U93244), *Nicotiana bentamiana* (NbCAT; accession no. EU998969), *Gossypium hirsutum* (GhCAT1; accession no. XP_016687939.1), *Solanum tuberosum* (StCAT2, accession no. KAH0685113.1), *Triticum turgidium ssp durum* (TdCAT1; Accession no. KP696753), *Aeogilops tauchii* (AetCAT1; accession no. XP_020164896.1), *Brachypodium dyctiosum* (BdCAT1, accession no. XP_003558892.1) and *Sorghum bicolor* (SbCAT1, accession no. XP_021304262.1).

a)

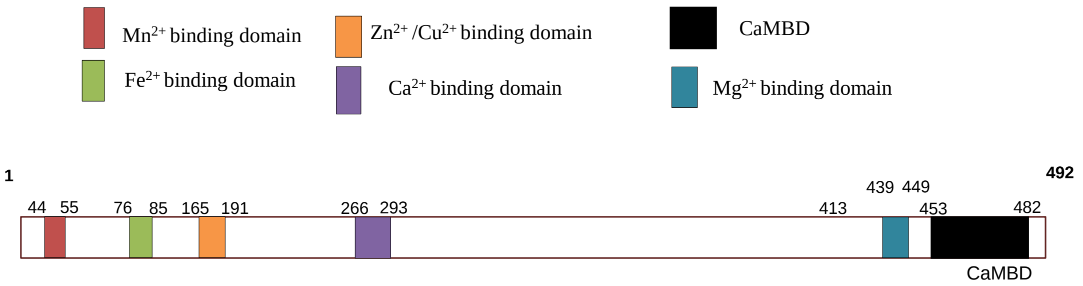

b)

```
..401 KHAPRYPIPS RTLNGRREKM VIEKENNFKQ PGERYRSMDP ARQERFINRW
..... 0000000000 0000000000 0000000000 0000000000 0000000000

..451 IDALSDPRLT HEIKAIWLSC WSQADKSLGQ KLASRLSSKP SM
..... 0000000000 0024444444 4444444444 4420000000 00
```

c)

CaMBD

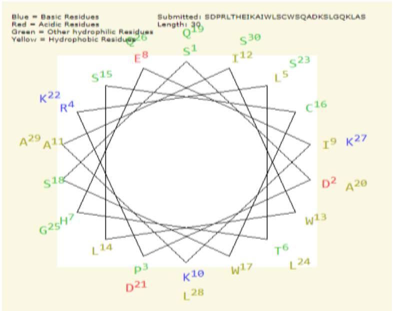

459 SDPRLTHEIKAIWLSCWAQADKSLGQKLAS **482**

d)

CaMBD (459-482)

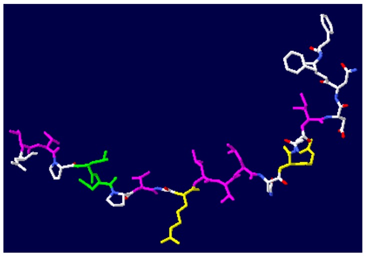

Supplemental Figure S6. TdCAT1 harbors a conserved CaMBD located at the C-terminal part of the protein. (a) Schematic presentation of TdCAT1 with identification of the conserved CaMBD represented in black. **(b)** Localization and Identification of the calmodulin binding domain as revealed by Calmodulin target Database. **(c)** Helical wheel projection of the motif for the Calcium dependent calmodulin binding domain (underlined sequences). Dashed lines separate proposed hydrophobic (h) and basic (+) faces of the NT and CT wheels. **(d)** 3D structure of CaMBD as revealed by spdv 1.4 program. The basic amino acids are represented bin yellow, the hydrophobic amino acids are represented in pink.

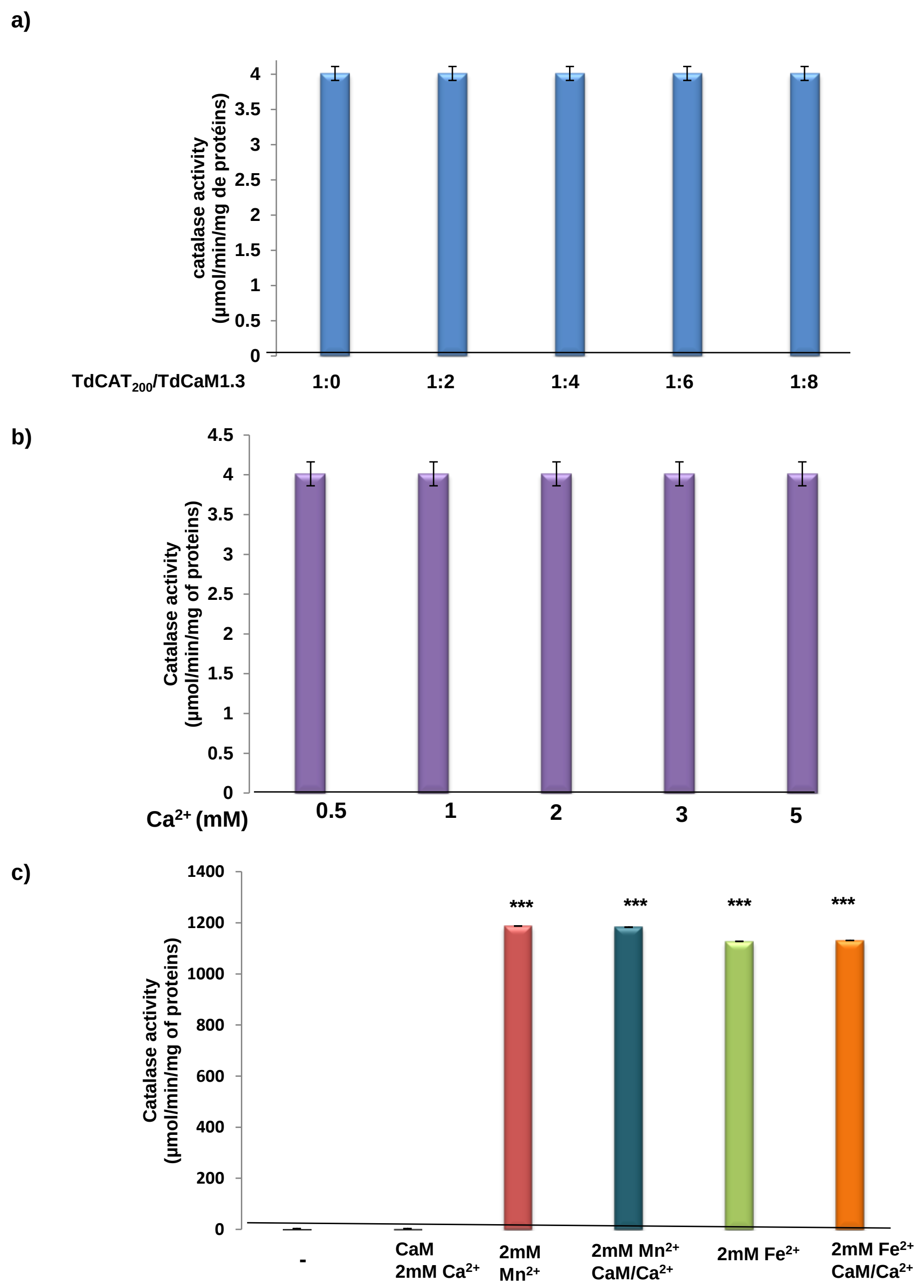

Supplemental Figure S7. The activity of His_TdCAT1$_{200}$ is not affected by TdCaM1.3/Ca^{2+} complex. (a) Catalase activities were measured according to the same conditions indicated before with a His_TdCAT1$_{200}$/TdCaM1.3 molar ratio ranging from 1:0 to 1:8 1:4. **(b)** TdCAT1$_{200}$ activity was not modified in presence of increasing concentrations of Calcium ranging from 0.5 to 5 mM. **(c)** Catalase activity of deleted form His-TdCAT$_{200}$ was stimulated in presence of Mn^{2+} and Fe^{2+} but not with CaM/Ca^{2+} complex. All data are mean values ± S.E of initial rate from three independent assays. (***) indicates value significantly different from the control. Statistical significance was assessed by applying the ANOVA test with <0.005.
